# Individual Behavior of Workers of the Formosan Subterranean Termite (Isoptera: Rhinotermitidae) on Consecutive Days of Tunnel Construction

**DOI:** 10.3390/insects3020367

**Published:** 2012-03-23

**Authors:** Mary L. Cornelius

**Affiliations:** United States Department of Agriculture, Agricultural Research Service, Southern Regional Research Center, 1100 Robert E. Lee Blvd. New Orleans, LA 70124, USA; E-Mail: mary.cornelius@ars.usda.gov; Tel.: +504-286-4449; Fax: +504-286-4419

**Keywords:** tunnel, behavior, social insects, subterranean termite, task allocation

## Abstract

This study examines the individual behavior of workers of the Formosan subterranean termite, *Coptotermes formosanus* Shirkai, on two consecutive days of tunnel construction. In each trial, a group of 30 termite workers was observed continuously during the first 60 min of construction of a new tunnel on two consecutive days. On each day, an average of 68% of individuals did not participate in tunnel construction, 19% spent <25 min tunneling, and 13% spent ≥25 min tunneling. There were specific individuals that did most of the work in the construction of new tunnels on both days. An individual that spent at least 25 min tunneling on Day 1 was significantly more likely to spend at least 25 min tunneling on Day 2 than individuals that spent <25 min tunneling on Day 1. When individuals were ranked based on the time spent tunneling on Day 1 and Day 2, there were individuals ranked as one of the top four excavators on both days in three of the four groups. These results indicate that there is evidence of task allocation by termite workers during the construction of a new tunnel.

## 1. Introduction

In social insects, such as ants and honey bees, elaborate systems of task allocation among castes have been documented extensively [1]. Division of labor occurs among workers of some termite species [2]. Black marching termites, *Hospitalitermes medioflavus* (Holmgren) and *H. umbrinus* (Haviland), forage in the open air and have three worker castes (minor, medium and major) that perform different tasks during food harvesting [3]. In the fungus growing termite, *Macrotermes bellicosus* (Smeathman), minor workers perform almost all exploration and construction, whereas major workers are recruited in mass only after a food source has been discovered [4]. 

Subterranean termites construct extensive networks of underground tunnels. There is less information about task allocation among subterranean termite workers due to their cryptic lifestyle. A study examining foraging and mound building behavior by workers of *Nasutitermes exitiosus* found that relatively few termite workers switched tasks. Marked foragers were twice as likely to be recaptured as foragers than as builders [5]. Crosland *et al.* [6] determined that older workers of the subterranean termite, *Reticulitermes fukienensis* Light carried out the highest frequency of all tasks investigated, including foraging-related tasks and care of eggs, larvae, and the queen. Yang *et al.* [7] found a positive correlation between excavation time by the Formosan subterranean termite workers, *Coptotermes formosanus* Shiraki, and workers antennal segment count which was used to determine worker age. However, excavation time of workers of the same age was highly variable, indicating that worker age is probably not the only determining factor for excavation activity. 

Numerous studies have examined the tunneling behavior of the Formosan subterranean termite [7,8,9,10,11,12,13,14,15,16,17,18,19,20,21]. Two studies specifically examined the role of individual workers in tunnel construction and found evidence of task allocation. In 4-h observations of 27 marked workers, only one or two specific individuals tunneled continuously, 59% of individuals tunneled for less than 1 h, and 16% of individuals did not tunnel [7]. When groups of 100 termites were presented with a single tunnel, only 16% of termites entered the tunnel and 20.6% of termites contributed more than 50% of total tunnel excavation [21]. Bardunias *et al.* [21] determined that specific individuals were responsible for most of the tunnel excavation and concluded that these key individuals may have the most influence on the orientation of the tunnel and the formation of branches.

When termites initiate construction of a new tunnel, only a single individual is able to enter the tunnel tip, and as the new tunnel gradually lengthens, more individuals become involved. Initially, only one or two individuals are involved in construction of a new tunnel. Tunnel width is related to the number of termites in the tunnel tip. Termites are more likely to widen the tunnel by engaging in lateral excavation when there are larger numbers present at the tunnel tip than when there are smaller numbers present at the tunnel tip [19].

Because only one or two individuals are involved in the construction of a new tunnel, it is possible that specific individuals play a role in the initiation of a new tunnel. The study presented here examined the tunneling behavior of marked individuals during the first hour of construction of a new tunnel on two consecutive days. The study examined whether specific individuals are more likely to tunnel than other individuals and whether their tunneling behavior on the second day can be predicted by their tunneling behavior on the first day. 

## 2. Experimental Section

### 2.1. Termite Collection

Termites were collected from field colonies in an urban forest, City Park, New Orleans, LA, where termites were being monitored in over 100 underground traps using cylindrical irrigation valve boxes (NDS, Inc, Lindsay, CA) that were buried in the ground and filled with blocks of wood (spruce, *Picea* sp.). The collected termites were maintained in the laboratory in 5.6- L covered plastic boxes containing moist sand and blocks of spruce (8 cm by 4 cm by 0.5 cm) until they were used in experiments. Termites were used within one month of collection.

### 2.2. Tunneling Assay

For each test, workers were uniquely marked on their dorsal abdomen with either a single color or a two color combination of eight different colors of enamel paint (Testor Corp., Rockford, IL). A group of 30 marked workers were introduced into a polystyrene, cylindrical screwtop container (4.5 cm high × 4.8 cm diameter). For each group of 30 marked workers, two trials were conducted on consecutive days using the same group of termites. There were two groups from each of two colonies. 

**Figure 1:**
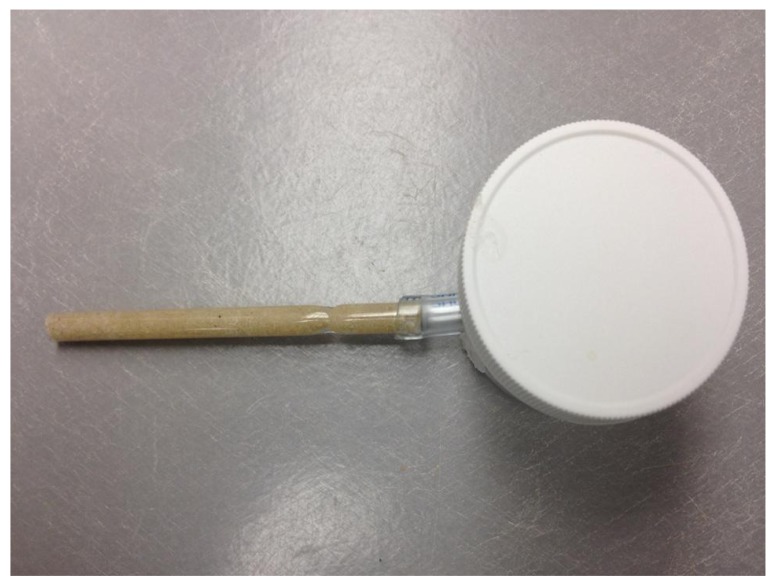
The testing apparatus for tunneling assays.

Termites were able to enter a 2 cm length piece of PVC tubing (0.6 cm I.D. by 1.0 cm O.D. by 0.2 cm Wall) (Nalgene, Rochester, NY) inserted through a hole on the ventral side of the container and sealed in place with hot glue from a glue gun. A glass tube (10 cm length, 1 cm diameter) was filled with sand (Play Sand, Quikrete, Atlanta GA) and thoroughly moistened with distilled water. An indentation (2 mm length by 2 mm width) was made on the top of the sand in the glass tube in order to decrease the length of time required for termites to initiate tunnel construction and to ensure that termites constructed a tunnel at the top of the tube so that individuals could be easily identified. The indentation allowed a single termite to enter the sand-filled tube and initiate tunnel construction. The glass tube was attached to the distal end of the PVC tubing (Figure 1). In each trial, termites were observed continuously until tunnel construction was initiated in the sand-filled glass tube and then termites were observed continuously for the first 60 min of tunnel construction. After 60 min, termites were removed from the container and tubing, placed in a glass petri dish with moist filter paper, and kept in a dark environmental chamber (28 °C, 90% RH) overnight. The following day, the same group of 30 marked termites was introduced into a new container with a new sand-filled glass tube attached and the experiment was repeated.

Tunneling behavior was defined by observing a termite picking up and moving sand particles. Before tunneling was initiated (the first time a termite picked up and moved sand particles), the number of times that a termite contacted the sand in the glass tube and left without tunneling was recorded. Once tunneling was initiated, the termites were observed continuously for 60 min and the time spent tunneling by each individual was recorded. The number of minutes spent tunneling by each individual on each day was determined for two consecutive days.

### 2.3. Statistical Analysis

The total time spent tunneling by marked individuals in both trials was divided into time periods (none, 1–10 min, 11–20, 21–40, 41–60, >60) and compared using a Kruskal-Wallis one-way ANOVA on ranks. Means were separated using Tukey’s test with ranked sums. The number of individuals that did not tunnel, spent <25 min, or ≥25 min tunneling was compared for each day separately and for each group separately using a Pearson chi-square test. Also, the number of termites that did not tunnel on either day, tunneled on only one day, or tunneled on both days were compared for each group using a Pearson chi-square test. The total time spent tunneling by individuals on both days for each colony was compared using a Mann-Whitney U Rank Sum. The time taken to start tunneling and the number of times a termite contacted the sand before tunnel construction was initiated for each colony was compared using a t-test. The total numbers of termites that spent time tunneling on Day 1 and Day 2 were compared using a Pearson Chi-square. 

## 3. Results and Discussion

Tunneling behavior of individuals was observed on consecutive days to determine if there was evidence for task allocation in tunneling behavior where key individuals did most of the work in the construction of a new tunnel on both days.

When comparing the total time spent tunneling by marked individuals on both days, the number of termites that tunneled different lengths of time was significantly different (Kruskal-Wallis: H = 17.48; df = 5; P = 0.004). There were significantly more termites that did not tunnel on either day than termites that spent either 41–60 min or >60 min tunneling (Tukey HSD Test: P ≤ 0.05) (Table 1). 

The number of individuals involved during the first hour of construction of a new tunnel was consistent for each trial. When the number of termites that did not tunnel, spent <25 min, or ≥25 min tunneling were compared for each day and each group separately, the proportion of individuals in each category was not significantly different in the different trials (Pearson chi-Square: 20.9; df = 14; P = 0.10) (Table 2). The proportion of individuals that participated in tunnel construction was similar for different groups of workers from the two colonies. An average of 68% of individuals did not participate in tunnel construction, 19% spent < 25 min tunneling, and 13% spent ≥ 25 min tunneling. These results were consistent with other studies examining tunneling behavior of Formosan subterranean termites that determined that a majority of individuals did not tunnel and that most of the tunnel construction was performed by a small number of individuals [7,21].

**Table 1:** Mean (±SE) number of termites that spent time tunneling for different time intervals when the number of minutes each termite spent tunneling on both days was combined. Trials were conducted on consecutive days with the same group of 30 workers with two groups from each colony.

Time Spent Tunneling	Mean (±SE) Number of Termites
None	14.0 ± 2.6a
1–10 min	5.0 ± 1.4ab
11–20 min	3.8 ± 0.9ab
21–40 min	3.8 ± 0.6ab
41–60 min	1.5 ± 0.3b
> 60 min	1.8 ± 0.5b

Kruskal-Wallis: H = 17.48; df = 5; P = 0.004.

**Table 2:** Number of termites in three categories of time spent tunneling (none, < 25 min, ≥25 min) in each 1-h tunneling trial conducted on consecutive days with the same group of 30 workers with two groups from each colony.

Colony-Group-Day	None	< 25 min	≥25 min
1-1-1	19	7	4
1-1-2	15	9	6
1-2-1	26	3	1
1-2-2	24	2	4
2-1-1	22	4	4
2-1-2	16	7	7
2-2-1	18	9	3
2-2-2	23	5	3
Mean (±SE)	20.4 ± 1.4	5.8 ± 0.9	3.9 ± 0.7

Pearson chi-Square: 20.9; df = 14; P = 0.10.

There was no significant difference in the total number of termites that spent time tunneling on Day 1 (35) compared with the total number of termites that spent time tunneling on Day 2 (43) (Pearson Chi-square: 1.2; df = 1; P = 0.27). There was no significant difference in the total number of minutes tunneled by termites on Day 1 (160 ± 40.2) compared with Day 2 (238.5 ± 55.1) (t-test: P = 0.29). Therefore, the disturbance involved in retesting the same individuals on a second day did not have an effect on the total number of termites tunneling, the total time spent tunneling, or the specific individuals involved in tunnel excavation. 

Also, there were no significant colony differences in the tunneling behavior of individuals. When the number of minutes spent tunneling by individuals on both days was combined, there was no difference in the total time spent tunneling by termites in Colony 1 (12.3 ± 3.0) and Colony 2 (14.2 ± 2.7) (Mann-Whitney U Rank Sum: P = 0.36). There was no significant difference in time taken to start tunneling by Colony 1 (44.3 ± 13.8) and Colony 2 (31.0 ± 12.6) (t-test: P = 0.50). There was no significant difference in number of times a termite contacted the sand before tunnel construction was initiated between Colony 1 (14.8 ± 7.1) and Colony 2 (2.3 ± 1.7) (t-test: P = 0.14). 

When the number of termites in three categories of time spent tunneling (none, one day only, both days) from both trials, was compared for each group, the proportion of individuals was not significantly different in the different groups (Pearson Chi-Square: 10.99; df = 6; P = 0.09) (Table 3). On average, 46% of individuals did not tunnel on either day, 43% of individuals tunneled on only one day, and 11% of individuals tunneled on both days. 

**Table 3:** Number of termites in three categories of time spent tunneling (none, one day only, both days) from both trials conducted on consecutive days with the same group of 30 workers with two groups from each colony.

Colony-Group	None	One Day Only	Both Days
1-1	9	16	5
1-2	21	8	1
2-1	12	14	4
2-2	14	13	3
Mean (±SE)	14.0 ± 2.6	12.8 ± 1.7	3.3 ± 0.8

Pearson Chi-Square: 10.99; df = 6; P = 0.09.

**Table 4:** Number of termites in three categories of time spent tunneling (none, < 25 min, ≥ 25 min) in each 1-h tunneling trial conducted on consecutive days for four groups of 30 workers, two groups for each colony.

Time Spent Tunneling by Marked Individuals on Day 1and Day 2			
(Row Percentages of Termite Numbers)			
Day 2			
Day 1	None	< 25 min	≥ 25 min
None	56 (72)	17 (22)	5 (6)
< 25 min	18 (78)	4 (17)	1 (4)
≥ 25 min	11 (58)	2 (10)	6 (32)

Pearson Chi-square: 12.0; df = 4; P = 0.02.

When the tunneling behavior of individuals was compared on the two days, the probability of termites tunneling on Day 2 was not significantly affected by their tunneling behavior on Day 1 (did not tunnel on either day: 56; tunneled on Day 1 only: 29; tunneled on Day 2 only: 22; tunneled on both days: 13) (Pearson chi square: 0.10; df = 1; P = 0.752) However, the outcomes were significantly different than expected when comparing whether individuals that either did not tunnel, spent < 25 min, or ≥ 25 min tunneling on Day 1 behaved similarly on Day 2 (Pearson Chi-square: 12.29; df = 4; P = 0.015). While 72% of individuals that did not tunnel on Day 1 did not tunnel on Day 2 and 6% spent ≥ 25 min tunneling, 32% of individuals that spent ≥ 25 min tunneling on Day 1 also spent ≥ 25 min tunneling on Day 2 (Table 4). Therefore, individuals that spent the most time tunneling on Day 1 were significantly more likely to spend the most time tunneling on Day 2. 

**Figure 2:**
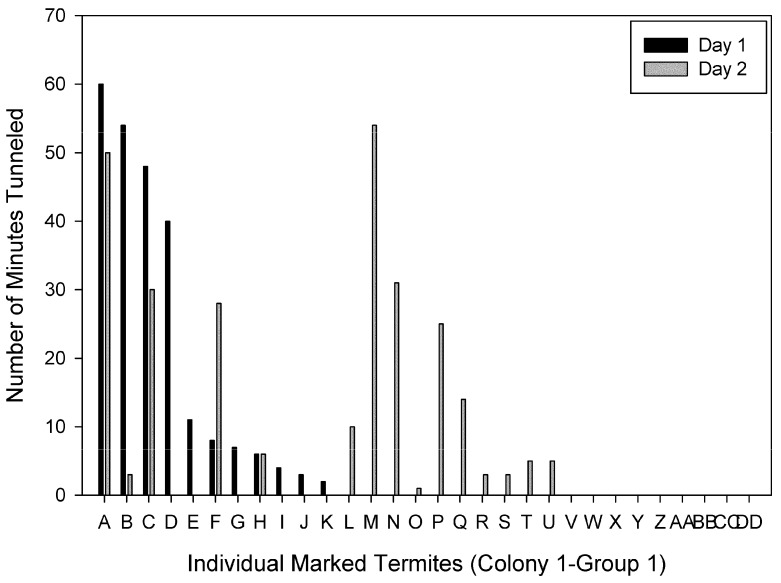
The number of minutes spent tunneling by each individual marked termite in Colony 1-Group 1 on Day 1 and Day 2.

**Figure 3:**
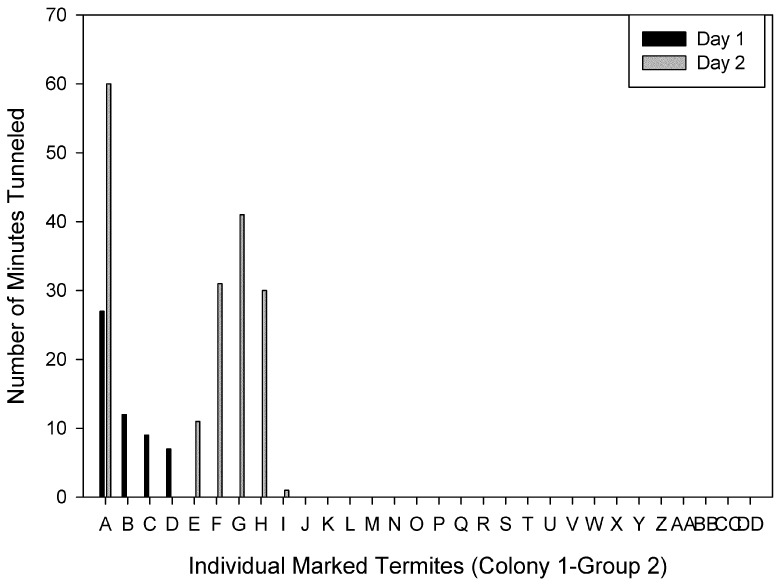
The number of minutes spent tunneling by each individual marked termite in Colony 1-Group 2 on Day 1 and Day 2.

**Figure 4:**
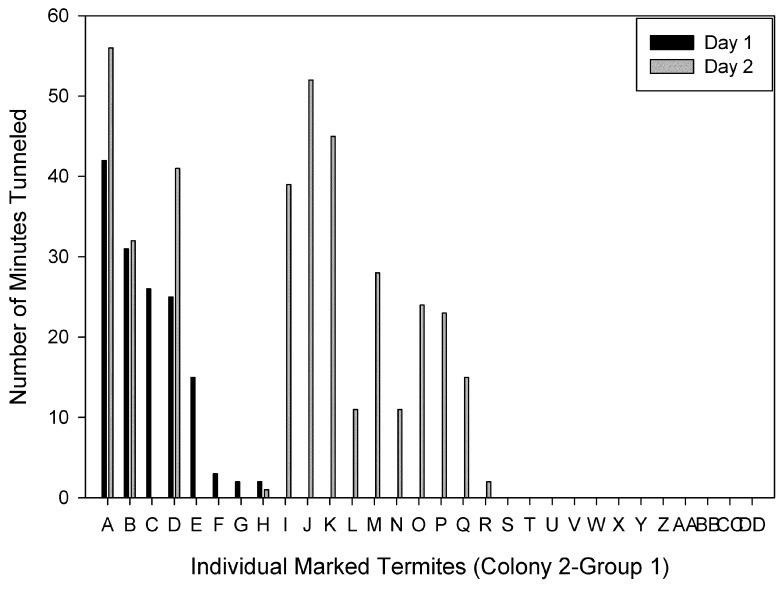
The number of minutes spent tunneling by each individual marked termite in Colony 2-Group 1 on Day 1 and Day 2.

**Figure 5:**
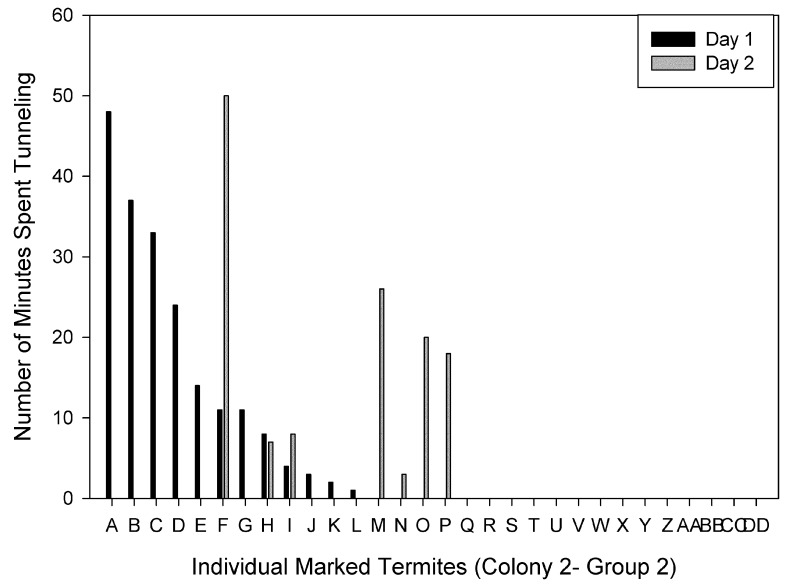
The number of minutes spent tunneling by each individual marked termite in Colony 2-Group 2 on Day 1 and Day 2.

Individuals were ranked based on the time spent tunneling on Day 1. Figures show individuals ranked from the most to the least time spent tunneling on Day 1 compared to the time each individual spent tunneling on Day 2 for each group. There were individuals ranked in the top four on both days in three of the four groups. For Colony 1-Group 1, two of the four primary excavators on Day 1 and 2 were the same individuals (Figure 2). The primary excavator on Day 1 spent a total of 110 min tunneling, and only four individuals spent a total of at least 50 min tunneling. For Colony 1-Group 2, the primary excavator on both days was the same individual, and 21 individuals did not tunnel at all on either day (Figure 3). For Colony 2- Group 1, two of the four primary excavators on Days 1 and 2 were the same individuals and one of the four primary excavators on Day 1 also spent > 30 min tunneling on Day 2 (Figure 4). There were four individuals that spent a total of at least 50 min tunneling. However, for Colony 2-Group 2, none of the four primary excavators on Days 1 and 2 were the same individuals (Figure 5). In this group, there was only one individual that spent a total of at least 50 min tunneling. 

This study identified specific individuals that were more likely than other individuals to initiate the construction of a new tunnel, and to spend the most time tunneling during the first 60 min of tunnel construction. These results suggest that there is task allocation in the construction of new tunnels. Only one or two individuals were involved during the first few minutes of tunnel construction. Additional individuals gradually got involved over the first hour. On average, four individuals did most of the work during the first hour of tunnel construction. An individual that spent at least 25 min tunneling on Day 1 was significantly more likely to spend at least 25 min tunneling on Day 2 than individuals that spent < 25 min tunneling on Day 1. Because the number of individuals involved in tunnel construction increases as the tunnel expands [19], further research is necessary to examine the role of individuals in tunnel construction as a new tunnel is expanded. 

## 4. Conclusions

Task allocation among termite workers has been well documented for open air foragers and fungus-growing termites [2]. Because of the cryptic nature of subterranean termites, the role of individual workers in tunnel construction has not been as well studied. This study demonstrated that specific individuals played a key role in tunnel construction on two consecutive days. 

Results from this study indicate that group size and arena size may affect termite behavior in tunneling assays designed to test the efficacy of soil termiticides. Lenz [22] suggested that laboratory assays conducted with small groups of termites may not achieve the same results as assays conducted with larger groups of termites. Individuals in smaller groups may need to perform basic tasks more frequently than any individual would do under natural conditions, resulting in higher energy expenditure and stress levels for members of smaller groups compared to individuals in larger groups. In tunneling assays, the number of termites entering the tunnel increases as the length of the tunnel increases. Also, the tunnel width increases as the number of termites attempting to reach the tunnel tip increases, causing increased lateral excavation [19]. When termites construct a network of tunnels in an arena, larger groups generally reach equilibrium at a greater tunnel volume than smaller groups [18]. However, only a small number of individuals are active during tunneling assays that require termites to tunnel a few centimeters. When groups of 100 termites were presented with a single tunnel, only 16% of individuals entered the tunnel [21]. In tunneling assays, increasing group size will not increase the number of individuals participating in tunnel excavation unless the distance tunneled increases as well.

In a study where *C. formosanus* workers were exposed to three termiticides at two population densities, termites tunneled significantly farther into treated soil at high population density than at low population density. However, there was no significant difference in mortality between the two population densities. Termites only tunneled an average of < 1 mm to 5.5 cm into treated soil [23]. When soil termiticides are evaluated by allowing groups of termites to tunnel a few centimeters into treated soil, only a small number of termites may actually become directly exposed to treated soil. Because non-repellent termiticides do not inhibit termites from tunneling into treated areas, these soil temiticides could be evaluated in assays designed to enable termites to construct a network of tunnels in treated soil. A greater percentage of termites would enter tunnels and participate in tunnel excavation when constructing a network of tunnels compared to a single tunnel.

In the current study, only a small number of individuals were involved in tunnel excavation, while most of the individuals were inactive. However, under natural conditions, individuals that are not involved in tunnel excavation may perform other tasks, such as colonizing new food sources, tunnel maintenance and repair, or brood care. Also, individuals that were not active during the 60 min observation period may act as a reserve work force [5]. Further research is necessary to determine whether individuals that are not involved in the excavation of new tunnels perform other tasks or act as a reserve labor force.

Increasing our understanding of the behavior of individuals in tunnel construction could eventually lead to a greater understanding of the physiological and behavioral processes that regulate termite tunneling behavior, termite sociality, and behavioral polyphenism. This knowledge could be used in the development of novel methods of termite control. 
